# DJ-1 Is a Redox-Dependent Molecular Chaperone That Inhibits α-Synuclein Aggregate Formation

**DOI:** 10.1371/journal.pbio.0020362

**Published:** 2004-10-05

**Authors:** Shoshana Shendelman, Alan Jonason, Cecile Martinat, Thomas Leete, Asa Abeliovich

**Affiliations:** **1**Departments of Pathology and Neurology, Center for Neurobiology and Behavior, and Taub Institute, Columbia University, College of Physicians and SurgeonsNew York, New YorkUnited States of America

## Abstract

Parkinson's disease (PD) pathology is characterized by the degeneration of midbrain dopamine neurons (DNs) ultimately leading to a progressive movement disorder in patients. The etiology of DN loss in sporadic PD is unknown, although it is hypothesized that aberrant protein aggregation and cellular oxidative stress may promote DN degeneration. Homozygous mutations in *DJ-1* were recently described in two families with autosomal recessive inherited PD (Bonifati et al. 2003). In a companion article (Martinat et al. 2004), we show that mutations in DJ-1 alter the cellular response to oxidative stress and proteasomal inhibition. Here we show that DJ-1 functions as a redox-sensitive molecular chaperone that is activated in an oxidative cytoplasmic environment. We further demonstrate that DJ-1 chaperone activity in vivo extends to α-synuclein, a protein implicated in PD pathogenesis.

## Abstract 2

The interaction of the proteins DJ-1 and α- synuclein described here may be important for understanding the molecular basis of Parkinson's disease

## Introduction

Parkinson's disease (PD) is a progressive movement disorder that is characterized pathologically by the relatively selective degeneration of midbrain DNs and the presence of prominent intracytoplasmic neuronal inclusions, termed Lewy bodies (Dauer and Przedborski 2003). The identification of several genes that underlie familial forms of primary parkinsonism has allowed for the molecular dissection of mechanisms of dopamine neuron (DN) survival. Autosomal dominant mutations in α-synuclein (αSyn) lead to a rare familial form of primary Parkinsonism (Polymeropoulos et al. 1997), and there is evidence that these mutations generate toxic, abnormal protein aggregates (Goldberg and Lansbury 2000) and proteasomal dysfunction (Rideout et al. 2001). Of note, Lewy body inclusions are particularly enriched for αSyn (Spillantini et al. 1998) and neurofilament protein subunits (Trojanowski and Lee 1998). Mutations in a second gene, *Parkin,* lead to autosomal recessive primary Parkinsonism (Hattori et al. 2000). Parkin is a ubiquitin ligase that appears to participate in the proteasome-mediated degradation of several substrates (Staropoli et al. 2003).

Homozygous mutations in a third gene, *DJ-1,* were recently associated with autosomal recessive primary Parkinsonism (Bonifati et al. 2003). *DJ-1* encodes a ThiJ domain protein of 189 amino acids that is broadly expressed in mammalian tissues (Nagakubo et al. 1997). Interestingly, DJ-1 was independently identified in a screen for human endothelial cell proteins that are modified with respect to isoelectric point in response to sublethal doses of paraquat (Mitsumoto and Nakagawa 2001; Mitsumoto et al. 2001), a toxin which generates reactive oxygen species (ROS) within cells and has been associated with DN toxicity (McCormack et al. 2002). Gene expression of a yeast homolog of DJ-1, YDR533C, is upregulated in response to sorbic acid (de Nobel et al. 2001), an inducer of cellular oxidative stress. These data suggest a causal role for DJ-1 in the cellular oxidative stress response.

ThiJ domain proteins are highly conserved and have been associated with several functions including protease and chaperone activities (Halio et al. 1996; Du et al. 2000). The crystal structure of DJ-1 demonstrates the presence of a highly conserved nucleophile elbow-like domain at cysteine 106, but the relative position of this residue differs from that of a structurally related ThiJ protease, PH1704, and does not appear to be permissible for proton transfer and protease catalysis (Wilson et al. 2003). Furthermore, DJ-1 forms an asymmetric homodimer with a prominent carboxy-terminal helical region present at the dimerization interface, which appears to limit access to the nucleophile elbow-like domain (Huai et al. 2003; Lee et al. 2003; Wilson et al. 2003).

DJ-1 displays significant homology to the carboxy-terminal domain of the Escherichia coli HPII catalase, as both proteins are divergent members of the type I glutamine amidotransferase family. Interestingly, the carboxy-terminal DJ-1 homology domain of HPII catalase lacks catalase activity, but rather appears to function as a chaperone in the correct folding of the catalytic core of the protein, and in thermal enzyme stability (Chelikani et al. 2003). Taira et al. (2004) recently reported that purified DJ-1 harbors catalase activity, and that overexpression of DJ-1 by transfection of neuroblastoma tumor cells inhibits the accumulation of ROS. In contrast, analysis of DJ-1-deficient cells (Martinat et al. 2004) revealed that such cells display an apparently normal initial accumulation of ROS, indicating that DJ-1 likely functions in a protective role downstream of ROS insult. Consistent with this, DJ-1-deficient cells are predisposed to apoptotic death in the context of oxidative stress (Martinat et al. 2004).

Here we demonstrate that DJ-1 functions as a redox-regulated molecular chaperone that is activated in an oxidizing environment. DJ-1 chaperone activity extends in vivo to αSyn, a protein that has been implicated in PD pathogenesis. DJ-1 activity is abrogated by the L166P mutation, associated with primary Parkinsonism, as a consequence of defective dimerization and reduced stability.

## Results

### DJ-1 Lacks Apparent Protease and Antioxidant Activities In Vitro

DJ-1 homologs have been reported to harbor protease (Halio et al. 1996; Du et al. 2000; Lee et al. 2003) and amidotransferase activities (Horvath and Grishin 2001). However, crystal structure analyses of DJ-1 suggest that this protein may not retain such catalytic activities (Honbou et al. 2003a; Huai et al. 2003; Lee et al. 2003; Tao and Tong 2003; Wilson et al. 2003). Consistent with this, purified DJ-1 preparations failed to display in vitro protease activity toward a variety of synthetic or natural substrates, and, similarly, DJ-1 lacked antioxidant (Table S1) or catalase activities (Figure S1) in vitro. Furthermore, cells deficient in DJ-1 appear unaltered in the initial accumulation of ROS in the context of acute oxidative stress (Martinat et al. 2004).

### DJ-1 Is a Redox-Dependent Molecular Chaperone

**Figure 1:**
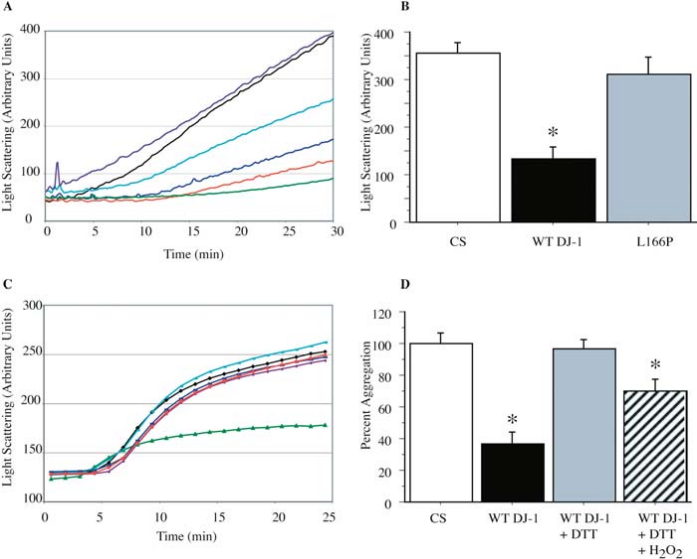
DJ-1 Is a Redox-Dependent Molecular Chaperone (A) Aggregation of CS was monitored at 43 °C after addition of either 0.8 μM CS alone (black), or along with 8.0 μM RNase A (purple), 0.5 μM DJ-1 (aqua), 2.0 μM DJ-1 (blue), 4.0 μM DJ-1 (red), or 2.0 μM Hsp27 (green). (B) Aggregation of 0.8 μM CS after 30 min at 4 °C (unfilled bar) is inhibited by 4.0 μM WT DJ-1 (black bar) but not 4.0 μM L166P mutant DJ-1 (gray bar). Data are shown as the mean ± SEM and were analyzed by ANOVA with Fisher's post-hoc test. * *p* < 0.05. (C) Aggregation of insulin (26 μM) B chains induced by 20 mM DTT at 25 °C. Insulin alone (black) or in the presence of 4.0 μM RNase A (purple), 0.5 μM DJ-1 (aqua), 2.0 μM DJ-1 (blue), 4.0 μM DJ-1 (red), or 2.0 μM Hsp27 (green). (D) CS thermal aggregation (unfilled bar) is suppressed by 4 μM DJ-1 (black bar), but chaperone activity is abrogated upon incubation of DJ-1 with 0.5 mM DTT for 10 min at 4 °C (gray bar). Further treatment of DTT-reduced DJ-1 with 10 mM H_2_O_2_ for 10 min at 4 °C leads to reactivation of CS suppression (hatched bar). Data are shown as the mean ± SEM and were analyzed by ANOVA with Fisher's post-hoc test. * *p* < 0.05.

Every organism responds to ROS and other toxic environmental stresses by overexpressing a highly conserved set of heat shock proteins (Hsps), many of which function as molecular chaperones to assist other proteins in folding. Hsp31, an E. coli ThiJ domain protein*,* has been shown to function as a molecular chaperone in vitro (Sastry et al. 2002; Malki et al. 2003). We hypothesized that DJ-1 may similarly function as a protein chaperone to protect cells from ROS. DJ-1 chaperone activity was quantified in the suppression of heat-induced aggregation of citrate synthase (CS) and glutathione S-transferase (GST), two well-characterized protein chaperone assays. These proteins lose their native conformation and undergo aggregation during incubation at 43 °C and 60 °C, respectively. Addition of 0.5–4.0 μM polyhistidine (His)-tagged DJ-1 was found to effectively suppress the heat-induced aggregation of 0.8 μM CS (Figure 1A). The chaperone activity was independent of the His tag used for purification, as cleavage and removal of the His tag did not alter DJ-1 chaperone function (unpublished data). DJ-1 chaperone activity is comparable to that of a well-described small cytoplasmic chaperone, human Hsp27. In contrast, RNase A failed to demonstrate chaperone activity and served as a negative control. Interestingly, the Parkinsonism-associated L166P DJ-1 mutation abrogated chaperone activity relative to the wild-type (WT) protein (Figure 1B).

DJ-1 similarly functioned as a molecular chaperone in the context of the heat-induced aggregation of GST (see Figure S1). In contrast, DJ-1 failed to display activity in a third chaperone assay, aggregation suppression of reduced insulin (Figure 1C). Reduction of the disulfide bonds between the A and B chains of insulin with dithiothreitol (DTT) leads to aggregation of the B chains. Hsp27 effectively inhibited the aggregation of insulin in the presence of 20 mM DTT, whereas neither DJ-1 nor the negative control protein RNase A displayed chaperone activity in this assay. As the insulin aggregation assay is performed in a reduced environment, we hypothesized that DJ-1 chaperone activity may be redox regulated. Interestingly, such a redox switch in a molecular chaperone has been described in Hsp33 (Jakob et al. 1999), a dimeric bacterial Hsp unrelated to DJ-1.

To test the redox regulation of DJ-1, we assayed chaperone activity in the CS aggregation assay in the presence or absence of the reducing agent DTT. DJ-1 chaperone activity in the CS aggregation assay was completely abrogated by preincubation of DJ-1 with 0.5 mM DTT in aggregation buffer for 10 min at 4 °C (Figure 1D). DTT did not significantly alter CS aggregation in the absence of DJ-1 and did not modify suppression of CS aggregation by Hsp27 (unpublished data). To further test whether redox regulation might govern DJ-1 chaperone activity, reactivation studies using reduced DJ-1 were performed. DTT-reduced DJ-1 was incubated with H_2_O_2_ (10 mM in aggregation buffer for 10 min at 4 °C followed by dialysis against aggregation buffer for 2 h), and subsequently chaperone activity was measured in the CS thermal aggregation assay. H_2_O_2_ effectively reactivated the chaperone activity of DTT-treated DJ-1 (Figure 1D). This was not an indirect effect of residual H_2_O_2_ on CS aggregation, as H_2_O_2_ treatment of CS increased aggregation (unpublished data). These results suggest that redox regulation of DJ-1 is reversible and is regulated by the redox environment.

Molecular chaperones typically display marked stability to thermal stress (Sastry et al. 2002). Consistent with this, the ultraviolet-circular dichroism (CD) spectrum of WT DJ-1 is consistent with a well-folded protein, and thermal denaturation of WT DJ-1 revealed a cooperative thermal unfolding transition at approximately 75 °C (see Figure S1). In contrast, the CD spectrum of the DJ-1 L166P mutant protein is typical of a partially unfolded polypeptide, suggesting that the L166P mutation causes a significant loss of helical structure. The mutant protein does not exhibit a thermal unfolding transition in the range studied (0–90 °C).

### DJ-1 Inhibits the Generation of αSyn Aggregates

**Figure 2:**
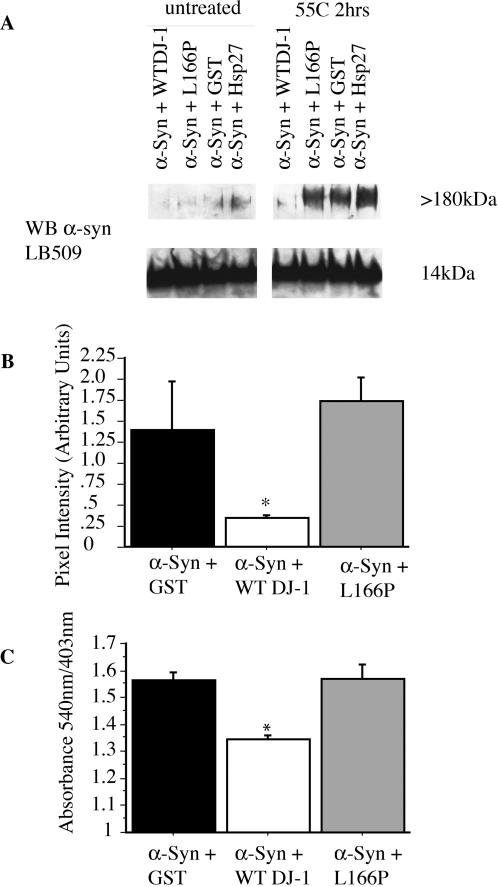
DJ-1 Inhibits Formation of αSyn Protofibrils and Fibrils In Vitro (A) Purified αSyn (200 μM) was incubated for 2 h at 55 °C in the presence of WT DJ-1, L166P mutant DJ-1, GST, or Hsp27 (all at 100 μM). WT DJ-1 inhibits accumulation of αSyn protofibrils in vitro, while L166P mutant DJ-1, GST, and Hsp27 do not. (B) Suppression of αSyn protofibril formation by WT DJ-1 (in triplicate) was quantified as compared to GST (as a negative control) and mutant L166P DJ-1. Data are shown as the mean ± SEM and were analyzed by ANOVA with Fisher's post-hoc test. * *p* < 0.05. (C) Purified αSyn (200 μM) was incubated for 1 wk at 37 °C in the presence of WT DJ-1, L166P mutant DJ-1, or GST (all at 100 μM). WT DJ-1 inhibits formation of mature Congo red–positive αSyn fibrils. Data are shown as the mean ± SEM and were analyzed by ANOVA with Fisher's post-hoc test. * *p* < 0.05.

We extended the analysis of DJ-1 chaperone function to a candidate DJ-1 substrate, αSyn (Figure 2). The aggregation of αSyn has been implicated in familial and sporadic forms of PD, as mutations associated with autosomal dominant familial primary Parkinsonism alter the propensity of αSyn to aggregate (Conway et al. 2000a), and as αSyn fibrils are a major constituent of the Lewy body intracytoplasmic inclusions that typify PD pathology (Spillantini et al. 1997). In vitro, monomeric αSyn is disordered or “natively unfolded” in dilute solution (Weinreb et al. 1996). Incubation of purified WT human αSyn for 2 h at 55 °C results in the generation of high molecular weight multimers that likely represent protofibrils (Figure 2A and 2B) (Volles et al. 2001; Gosavi et al. 2002). This treatment does not result in formation of mature amyloid fibrils, as determined by Congo red staining (see Figure S1). WT DJ-1 effectively inhibits the formation of soluble αSyn protofibrils at a molar ratio of 1:2 (DJ-1: αSyn). In contrast, L166P mutant DJ-1, GST, and Hsp27 (Figure 2A and 2B) failed to inhibit the generation of αSyn protofibrils.

αSyn protofibrils have been shown to be an intermediate in the formation of mature amyloid fibrils. Because DJ-1 chaperone activity is effective at inhibiting the accumulation of αSyn protofibrils, we sought to investigate the role of this activity in the generation of Congo red–positive mature fibrils. Congruently, WT DJ-1 inhibited formation of Congo red-positive αSyn fibrils, while L166P DJ-1 and GST did not (Figure 2C). Thus, DJ-1 seems to inhibit formation of αSyn fibrils by preventing formation of αSyn high molecular weight oligomers, or protofibrils. Interestingly, PD-associated clinical mutations in αSyn appear to accelerate oligomerization and protofibril formation (Volles et al. 2001).

### DJ-1 Chaperone Activity In Vivo

**Figure 3:**
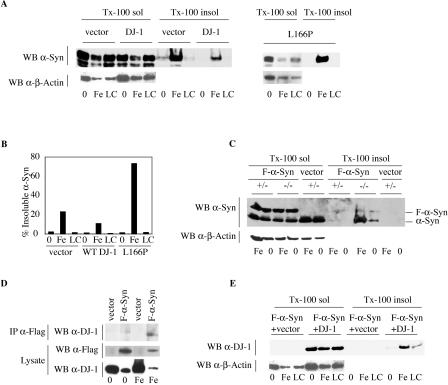
Overexpression of WT DJ-1 Inhibits Aggregation of αSyn In Vivo (A) CAD murine neuroblastoma cells were transfected with Flag-αSyn along with WT DJ-1, L166P clinical mutant, or vector alone, and were differentiated in vitro via serum withdrawal. Cells were subsequently treated with 2 mM FeCl_2_ (Fe), 5 μM lactacystin (LC), or media alone (0). Triton X-100-soluble (Tx-100 sol) and Triton X-100-insoluble (Tx-100 insol) fractions were analyzed by Western blotting. Upon FeCl_2_ treatment, αSyn accumulates in the Triton X-100-insoluble fraction, and accumulation of insoluble αSyn is inhibited by overexpression of WT DJ-1 (left) but not the L166P clinical mutant (right). (B) Triton X-100-insoluble αSyn as quantified by NIH Image J of a Western blot (from [A]). (C) Heterozygous (+/–) and DJ-1 deficient (–/–) ES cells were differentiated using the embryoid body protocol. Cells were transfected with Flag-αSyn (F-αSyn), and, after 48 h, treated with 2 mM FeCl_2_ or with media alone for 18 h. Cell lysates were analyzed by Western blotting for αSyn or β-actin. In the Triton X-100-soluble fraction (Tx-100 sol), DJ-1 accumulated to a similar extent in the knockout and control cells. In contrast, αSyn accumulation in the insoluble pool (Tx-100 insol) was detectable only in the knockout cells, and this was further promoted by FeCl_2_ treatment. (D) CAD cells transfected with Flag-αSyn (F-αSyn) along with WT DJ-1 (or vector alone) were treated with 2 mM FeCl_2_ or media alone for 18 h. Triton X-100-soluble cell lysates were immunoprecipitated with a mouse monoclonal antibody for the Flag epitope and Western blotted for DJ-1. FeCl_2_ treatment induces association of Flag-αSyn with WT DJ-1. Lysates represent 20% input of the immunoprecipitation (IP α-Flag). The Triton X-100 soluble pool of DJ-1 is reduced by αSyn overexpression (but not vector control), particularly in the context of FeCl_2_ treatment (bottom). (E) DJ-1 colocalizes with αSyn in the Triton X-100-insoluble fraction upon FeCl_2_ treatment. The Western blot from (A) was stripped and reprobed for DJ-1.

We sought to investigate the chaperone activity of DJ-1 toward αSyn in vivo. αSyn has been shown to form aggregates that consist of both protofibrils and mature amyloid fibrils in the context of oxidative stress (such as FeCl_2_ treatment [Lee and Lee 2002; Lee et al. 2002]) in neuroblastoma cells. We evaluated the activity of DJ-1 overexpression on αSyn aggregation in this tissue culture model system. Briefly, CAD murine neuroblastoma cells (Staropoli et al. 2003) were transfected with Flag epitope-tagged αSyn (Flag-αSyn), differentiated via serum withdrawal, and exposed to FeCl_2_ (2 mM) for 18 h. Treatment with FeCl_2_ induced accumulation of αSyn in the Triton X-100-insoluble fraction, which has been shown to correlate with αSyn protofibrils (Lee and Lee 2002). Overexpression of WT DJ-1, but not L166P clinical mutant DJ-1, significantly inhibited the accumulation of Triton X-100-insoluble αSyn (Figure 3A and 3B). DJ-1 overexpression did not alter the accumulation (Figure 3A) or half-life of soluble αSyn, as determined by pulse-chase kinetic analysis (Figure S2). Thus, DJ-1 overexpression is sufficient to inhibit the formation of αSyn aggregates in vivo, consistent with the in vitro analysis.

To investigate whether DJ-1 is necessary to inhibit αSyn aggregation in vivo, we utilized *DJ-1* “knockout” embryonic stem (ES) cells, which display increased sensitivity to oxidative stress. *DJ-1* homozygous knockout or control heterozygous ES cells (heterozygous cells were used as controls because they were the source of the knockout subclones) were differentiated in vitro using the embryoid body protocol (Martinat et al. 2004) and transfected with Flag-αSyn or control vector. Upon differentiation, both endogenous αSyn and transfected Flag-αSyn are accumulated to a similar extent in the soluble fraction of knockout and control cell lysates, as determined by Western blotting with an antibody for αSyn. In contrast, DJ-1-deficient cells (but not control cells) additionally accumulate Triton X-100-insoluble αSyn (both endogenous αSyn and transfected Flag-αSyn), which likely corresponds to protofibril aggregates (Lee and Lee 2002). As predicted, FeCl_2_ treatment further promoted the accumulation of insoluble αSyn in DJ-1-deficient cells but not in control heterozygous cells (Figure 3C). Interestingly, transfection of Flag-αSyn into undifferentiated knockout or control ES cells in the presence or absence of FeCl_2_ treatment did not lead to the accumulation of insoluble Flag-αSyn (see Figure S2), consistent with a prior study suggesting a role for neuronal differentiation in the generation of insoluble αSyn aggregates (Lee et al. 2002).

To investigate the mechanism of DJ-1 activity toward αSyn, we performed coimmunoprecipitation experiments on untreated and FeCl_2_-treated CAD cells transfected with DJ-1 and Flag-αSyn (or control vector) as above. Triton X-100-soluble cell lysates were immunoprecipitated with a mouse monoclonal antibody for the Flag epitope, and Western blots were probed with a rabbit polyclonal antibody for DJ-1. DJ-1 failed to interact with Flag-αSyn in the absence of pretreatment with FeCl_2_, but an association was evident in FeCl_2_-treated cell lysates (Figure 3D). Furthermore, overexpression of αSyn (but not vector control) leads to a reduction in the soluble pool of DJ-1, particularly in the context of FeCl_2_ treatment, indicating that DJ-1 additionally associates with an insoluble fraction of αSyn (Figure 3D, bottom panel). Consistent with this, we found that a significant fraction of DJ-1 protein localizes to the insoluble fraction upon FeCl_2_ treatment (Figure 3E) in cells that have been cotransfected with Flag-αSyn.

**Figure 4:**
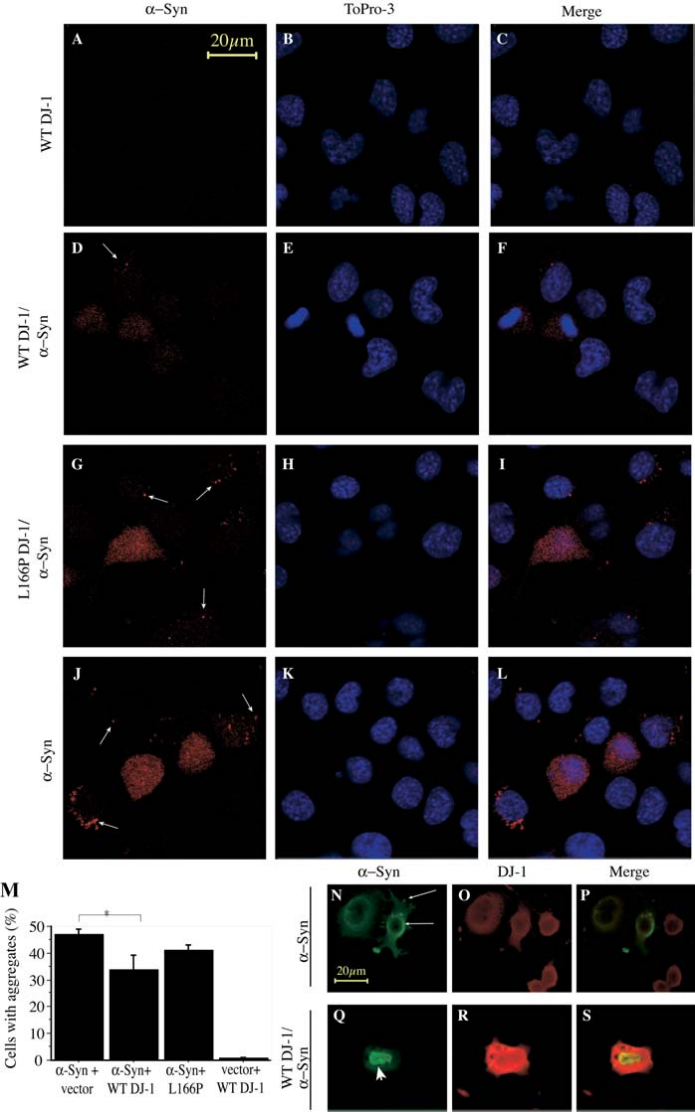
DJ-1 Inhibits Formation of αSyn Intracytoplasmic Inclusions (A–L) CAD murine neuroblastoma cells were transfected with WT DJ-1 (A–F), L166P DJ-1 (G–I) or vector control (J–L), along with Flag-αSyn (D–L) or vector control (A–C) and differentiated in vitro by serum withdrawal for 72 h. Cells were fixed and stained with a mouse monoclonal antibody for αSyn and ToPro3, a nuclear dye, and images were obtained by confocal microscopy. Transfection of Flag-αSyn induced formation of intracytoplasmic inclusions (arrows). Scale bar, 20 μm. (M) Quantification of cells with inclusions was performed on ten random images from each of three wells per condition. Images were quantified by an observer blinded to the experiment. A significantly lower percentage of cells harbor inclusions in the context of WT DJ-1 overexpression. Aggregation is expressed as the percentage of cells containing αSyn aggregates per frame. Total cell number per frame, as determined by ToPro3 staining, did not differ significantly (Figure S3). Data are shown as the mean ± SEM, and were analyzed by ANOVA with Fisher's post-hoc test. * *p* < 0.05. (N–S) Cells were fixed and stained with a monoclonal antibody for αSyn and a polyclonal antibody that recognizes both transfected human DJ-1 and endogenous murine DJ-1. DJ-1 does not appear to colocalize with the αSyn aggregates. Scale bar, 20 μm.

To further evaluate αSyn aggregation, we performed immunohistochemical analyses of CAD cells transfected with αSyn along with DJ-1 or control vector (Figure 4). Overexpression of αSyn in neuroblastoma cells induces the formation of visible cytoplasmic aggregates (Lee and Lee 2002) (Figure 4J–4L). Additional overexpression of WT DJ-1 significantly decreased the number of cells containing αSyn aggregates (Figure 4D–4F and 4M), whereas the L166P DJ-1 mutant fails to do so (Figure 4G–4I and 4M). However, DJ-1 does not appear to colocalize with αSyn aggregates, suggesting that DJ-1 functions at an early step in the formation of mature aggregates (Figure 4N–4S).

**Figure 5:**
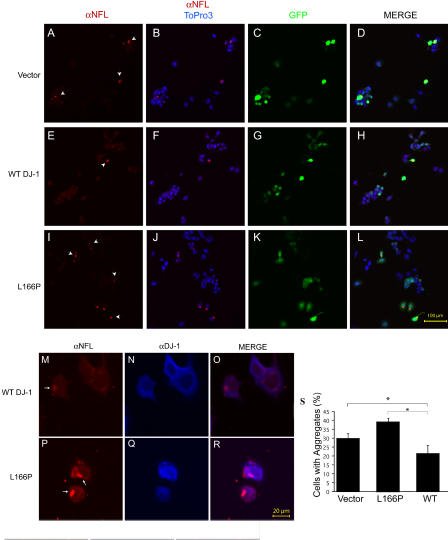
DJ-1 Inhibits Formation of NFL Intracytoplasmic Inclusions (A–L) CAD cells were transfected with an aggregation-prone mutant NFL (Q333P) plasmid, as well as WT human DJ-1 plasmid (that also harbors GFP; E–H), L166P mutant DJ-1 (that also harbors GFP; I–L), or control GFP vector (A–D). After 72 h in culture, cells were fixed and stained with a mouse monoclonal antibody for NFL and ToPro3, a nuclear dye. Scale bar, 100 μm. (M–R) CAD cell transfectants, as above, were fixed and stained with a polyclonal antibody for NFL (Perez-Olle et al. 2002) along with a mouse monoclonal antibody specific for the transfected human DJ-1. Scale bar, 20 μm. (S) Quantification of CAD cell NFL aggregates was performed using confocal microscopy. Images from tenrandomly selected fields in each of three wells were quantified for the presence of aggregates for each condition and presented as a percentage of total cells per field. Total cell number was determined by ToPro3 nuclear staining and did not differ significantly (Figure S3). Data are shown as the mean ± SEM and were analyzed by ANOVA with Fisher's post-hoc test. * *p* < 0.05.

In a separate set of experiments, we assayed the ability of DJ-1 to inhibit aggregation of a second substrate, neurofilament light subunit (NFL). Overexpression of a mutant form of human NFL, Q333P, by transient transfection of CAD murine neuroblastoma cells, leads to the accumulation of intracytoplasmic inclusions (Perez-Olle et al. 2002). Co-overexpression of WT DJ-1 along with mutant NFL significantly inhibited the accumulation of NFL inclusions (Figure 5), whereas overexpression of the L166P Parkinsonism-associated mutant form of DJ-1 with NFL failed to inhibit the accumulation of aggregates. Coimmunostaining for DJ-1 and NFL indicated that DJ-1 does not colocalize with the NFL inclusions (Figure 5M–5R). DJ-1 did not appear to alter the expression of NFL (Figure S3). These data are consistent with our analysis of DJ-1 chaperone activity toward αSyn and indicate that DJ-1 harbors chaperone activity toward a range of substrates in vivo.

### DJ-1 Function Requires Cysteine 53

**Figure 6:**
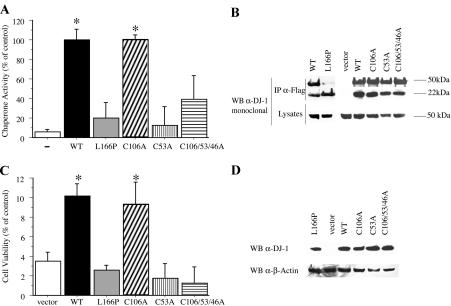
DJ-1 In Vitro Chaperone Activity and In Vivo Oxidative Stress Protection Activity Require Cysteine 53 but Not Cysteine 106 (A) DJ-1 cysteine-to-alanine mutants C106A, C53A, and a triple mutant that harbors mutations at all three cysteines in DJ-1 (C106A/C53A/C46A), as well as L166P, were tested for in vitro chaperone activity by CS aggregation suppression assay. (B) Self-association of DJ-1 cysteine mutants. Murine neuroblastoma CAD cells were transiently cotransfected with Flag-tagged human DJ-1 vectors (either WT or mutant) along with WT YFP-tagged human DJ-1. Lysates were immunoprecipitated with anti-Flag antibodies and probed by Western blotting with an antibody specific for human DJ-1. WT Flag-DJ-1, C106A DJ-1, C53A DJ-1, and C106A/C53A/C46A DJ-1 effectively coprecipitated WT GFP-DJ-1, whereas the L166P mutant Flag-DJ-1 failed to do so. Lysates represent 20% of the input for the immunoprecipitate; Flag-DJ-1 migrates at 22 kDa, and YFP-DJ-1 migrates at 50 kDa. (C) DJ-1-deficient ES cells were transiently transfected with vector alone, WT DJ-1, or DJ-1 cysteine mutants, and exposed to 10 μM H_2_O_2_ for 15 h followed by MTT assay. The viability of the cells in the absence of drug treatment was not altered by the expression of WT or mutant DJ-1). Data are shown as the mean ± SEM and were analyzed by ANOVA with Fisher's post-hoc test. * *p* < 0.05 (D) Expression levels of WT and mutant forms of DJ-1 were comparable as determined by Western blotting for human DJ-1 and β-actin.

The DJ-1 crystal structure suggests the presence of two highly reactive cysteines, cysteine 106 (Lee et al. 2003; Wilson et al. 2003) and cysteine 53 (Honbou et al. 2003b). To test whether reactive cysteines play a critical role in the function or regulation of DJ-1 activity, we mutagenized each cysteine in DJ-1 to alanine (Figure 6). Surprisingly, mutation of cysteine 106, at the predicted nucleophile elbow of DJ-1, does not alter the basal activity (Figure 6A) or the DTT sensitivity (See Figure S1) of DJ-1 chaperone function. In contrast, mutation of cysteine 53, which is present at the dimeric interface of DJ-1, completely abrogates chaperone activity. Similarly, mutation of all three cysteines in DJ-1 (cysteine 106, cysteine 53, and cysteine 47) leads to the loss of chaperone function. The cysteine mutations do not alter DJ-1 dimerization (Figure 6D) or the apparent stability of DJ-1 in vivo (unpublished data), unlike the L166P Parkinsonism-associated mutation.

DJ-1-deficient ES cells display increased sensitivity to oxidative stress, and this phenotype can be “rescued” by overexpression of WT DJ-1 but not PD-associated L166P mutant DJ-1 (Martinat et al. 2004). We further investigated the activity of the cysteine-mutant forms of human DJ-1 in vivo in the complementation of DJ-1-deficient ES cells. Cysteine 106–mutant DJ-1 robustly rescued DJ-1 knockout cells from H_2_O_2_ toxicity, consistent with the in vitro chaperone activity assay (Figure 6C). In contrast, cysteine 53 and the triple-cysteine mutant forms of DJ-1 failed to protect from H_2_O_2_ toxicity. These data support a role for cysteine 53–dependent chaperone activity in DJ-1-mediated ROS protection, and demonstrate a direct correlation between DJ-1 in vitro chaperone activity and cellular protection from oxidative stress. Our data are consistent with the prior observation that mutation of cysteine 53 to alanine abrogates the low–isoelectric point variant that is induced by oxidative stress (Honbou et al. 2003a).

## Discussion

We provide evidence that DJ-1 functions as a cytoplasmic redox-sensitive molecular chaperone in vitro and in vivo. This activity extends to αSyn and the neurofilament subunit NFL, proteins implicated in PD pathology. In a companion article (Martinat et al. 2004), we show that DJ-1 deficiency sensitizes cells to oxidative stress, leading to increased apoptosis in the context of an ROS burst. Taken together, our data strongly support the notion that DJ-1 functions as a redox-dependent protein chaperone to mitigate molecular insults downstream of an ROS burst. Oxidation-modified proteins have been shown to accumulate in the context of normal aging and PD, and may participate in the generation of protein aggregates in neurodegenerative disorders (Jenner 2003).

It is of interest to identify relevant in vivo substrates for DJ-1 activity in the context of DNs in PD. Our data suggest that DJ-1 activity extends to multiple targets, reminiscent of other small protein chaperones (Gusev et al. 2002), and consistent with this, DJ-1 activity is not ATP-dependent (unpublished data). Candidate substrates for DJ-1 chaperone activity in the context of PD include αSyn and neurofilament proteins, based on their presence in PD protein inclusions. Our data suggest that DJ-1 functions to suppress protein aggregates in the cytoplasm. It is possible that DJ-1 plays additional roles in the mitochondria or nucleus, as has been suggested (Bonifati 2003; Canet-Aviles 2004), although DJ-1 appears to remain localized diffusely in the cytoplasm with or without toxin treatment in our studies (see Figure S2).

Our data indicate that DJ-1 can suppress an early step in the formation of αSyn aggregates, the generation of high molecular weight oligomers (protofibrils). Interestingly, it has been suggested that such protofibrils, rather than the large fibrillar aggregates, may underlie αSyn toxicity in vivo (Volles et al. 2001). DJ-1 inhibits the aggregation of αSyn in differentiated cells in vivo, and loss of DJ-1 leads to increased accumulation of insoluble αSyn. DJ-1 appears to associate with αSyn in the Triton X-100-soluble fraction of FeCl_2_-treated lysates, and DJ-1 colocalizes with αSyn in the Triton X-100-insoluble fraction in the context of FeCl_2_ treatment. However, DJ-1 does not colocalize with the punctate protein aggregates visible by immunostaining in the case of either αSyn or NFL. This supports the notion that DJ-1 functions at an early step in the aggregation process, when the substrate protein may be misfolded, but has not yet formed a mature aggregate. We hypothesize that DJ-1 may promote the degradation of such misfolded proteins, either through the proteasome or through other cellular pathways such as chaperone-mediated autophagy.

A recent study investigated the chaperone activity of WT DJ-1 in vitro toward CS and concluded that redox regulation was not a significant factor (Lee et al. 2003). This is most likely a consequence of the use of only oxidizing conditions (0.5 mM H_2_O_2_) but not reducing conditions in the described chaperone assays (Lee et al. 2003). A second report failed to detect DJ-1 chaperone activity in vitro (Olzmann et al. 2003), but importantly, this study employed only reducing conditions in which DJ-1 chaperone activity is abrogated. In the present study we demonstrate that DJ-1 chaperone activity is inhibited by reducing conditions, and can be stimulated by oxidation. Thus, in the normal reducing environment of the cell, DJ-1 may be inactive. Production of ROS and alteration of the redox state of the cytoplasm may activate DJ-1 chaperone activity as a mechanism of coping with protein aggregation and misfolding.

We find that that the PD-associated L166P mutant DJ-1 fails to function as a molecular chaperone in vivo or in vitro. Consistent with this, in a companion article (Martinat et al. 2004), we show that this mutant fails to complement *DJ-1* knockout cells in vivo, even when overexpressed at artificially high levels (Martinat et al. 2004). Furthermore, the L166P mutant form fails to dimerize even when expressed at WT levels. Thus, although prior studies (Miller et al. 2003) and our analyses (unpublished data) have found that the L166P PD-associated DJ-1 mutation leads to decreased protein stability, it is apparent that even overexpression of the L166P mutant protein does not restore function. The L166P clinical phenotype is not due simply to reduced levels of DJ-1 protein, and, furthermore, we do not observe evidence of altered subcellular localization of the L166P mutant protein (Figure 4M-4R). Rather, our studies favor a model by which the pathological mechanism of this mutation is a consequence of altered structure and resultant loss of function.

Mutation of cysteine 53 in DJ-1 abrogates both chaperone and protective functions of this protein. Interestingly, cysteine 53 has previously been implicated as a reactive cysteine required for the in vivo modification of DJ-1 to a lower isoelectric point in response to oxidative stress (Honbou et al. 2003a), consistent with a role for such redox regulation in vivo. In contrast, cysteine 106, which has been reported to be sensitive to oxidative modification in vitro (Wilson et al. 2003), does not appear to be required for the in vitro and in vivo DJ-1 activities.

## Materials and Methods

### 

#### Cell culture and in vivo assays.

Undifferentiated ES cells, CAD neuroblastoma cells, and HeLa cells were cultured using standard techniques (Abeliovich et al. 2000; Staropoli et al. 2003). Transfections were performed using Lipofectamine 2000 (Life Technologies, Carlsbad, California, United States) for 18–36 h according to the manufacturer's instructions.

For in vivo αSyn aggregation assays, CAD cells were transfected with Flag-αSyn (pcDNA3) or DJ-1 (pCMS), and medium was replaced with medium without serum. Cells were cultured without serum to induce differentiation for 48 h post-transfection, at which time the medium was exchanged for medium alone or containing 2 mM FeCl_2_ and 5 μM lactacystin. Cells were treated with toxin for 18 h, then lysed or fixed with 4% PFA. Cell lysis was performed by resuspending cells in 50 mM Tris (pH 7.6), 150 mM sodium chloride, 0.2% Triton X-100, and protease inhibitor cocktail (Sigma, St. Louis, Missouri, United States). Cells were incubated on ice for 20 min and Triton X-100-soluble and -insoluble fractions were separated via centrifugation at 13,000 rpm for 15 min.

Quantification of CAD cell aggregates was performed using a Zeiss LSM Pascal confocal microscope (Zeiss, Oberkochen, Germany) with a 20× long working distance lens. Images were imported to NIH Image J for analysis. Images from tenrandomly selected fields in each of three wells were quantified for each condition. Cells containing at least one intracytoplasmic aggregate, independent of size or number per cell, were scored as positive for aggregates. This number was divided by the number of transfected cells per field, determined by GFP fluorescence.

#### ES cell culture and in vitro differentiation

Mouse ES cells were propagated and differentiated as described (Martinat et al. 2004). ES cells were differentiated via the embryoid body protocol. Cells were transfected with Flag-αSyn (pCMS) using Lipofectamine 2000 as per the manufacturer's instructions. 48 h post-transfection, cells were treated with 2 mM FeCl_2_ (or media alone) for 18 h.

#### Antibodies.

An anti-DJ-1 rabbit polyclonal antibody was generated against the synthetic polypeptide QNLSESPMVKEILKEQESR, which corresponds to amino acids 64–82 of the mouse protein. Antiserum was produced using the Polyquick polyclonal antibody production service of Zymed Laboratories (South San Francisco, California, United States). The antiserum was affinity purified on a peptide-coupled Sulfolink column (Pierce Biotechnology, Rockford, Illinois, United States) according to the manufacturer's instructions. Antibody was used at a dilution of 1:200 for immunohistochemistry and Western blotting as described (Staropoli et al. 2003). Immunohistochemistry was performed with a rabbit polyclonal antibody to DJ-1 (Martinat et al. 2004), TH (PelFreez, Rogers, Arizona, United States; dilution 1:1000), and a rabbit polyclonal antibody to GABA (Sigma; dilution 1:1000). Western blotting was performed using monoclonal antibody to DJ-1 (Stressgen Biotechnologies, San Diego, California, United States; dilution 1:1000), a monoclonal antibody to αSyn LB509 antibody (Zymed), and a monoclonal antibody to β-actin (Sigma; dilution 1:500). Mouse monoclonal antibody to NFL (Sigma; dilution 1:200) and rabbit polyclonal antibody to NFL (Perez-Olle et al. 2002). ToPro3 (Molecular Probes, Eugene, Oregon, United States; dilution 1:1000) was used as a nuclear dye.

#### Expression vectors.

*DJ-1* cDNA was PCR amplified from human liver cDNA (Clontech, Palo Alto, California, United States) and cloned into the expression vectors pET-28a (Novagen, Madison, Wisconsin, United States) or pcDNA3.1 (Invitrogen, Carlsbad, California, United States). Flag-DJ-1 and all described mutants were generated by PCR-mediated mutagenesis using standard techniques.

#### In vitro preparation of WT and mutant DJ-1.

His-tagged recombinant human WT or L166P DJ-1 was produced in E. coli BL21 cells induced with 1 mM IPTG for 4 h at 37 °C. Bacterial pellets were resuspended in 50 mM sodium phosphate (pH 6.8) and 300 mM sodium chloride, and lysed by sonication. Lysates were cleared by centrifugation at 20,000 × g for 20 min, and the supernatant was incubated with NTA-Ni-conjugated agarose resin for 1 h at 4 °C. The resin was subsequently washed five times with 20 resin volumes of lysis buffer containing 20 mM imidazole, and protein was eluted in five fractions of two resin volumes of lysis buffer containing 250 mM imidazole. Recombinant protein elutions were confirmed to be of > 99% purity by SDS-PAGE and colloidal Coomassie staining.

#### Aggregation assays.

CS aggregation was performed in 40 mM HEPES (pH 7.8), 20 mM potassium hydroxinde, 50 mM potassium chloride, and 10 mM ammonium sulfate, and monitored in a thermostat-controlled fluorescence spectrophotometer with excitation and emission wavelengths at 500 nm and slit widths at 2.5 nm. Insulin aggregation was performed as described (Giasson et al. 2000). CS, insulin, RNase A, and GST were obtained from Sigma; human Hsp27 was obtained from Stressgen.

αSyn protofibril and fibril formation assays were performed essentially as described (Uversky et al.). Briefly, protofibrils were formed by incubation of 200 μM WT synuclein with 100 μM DJ-1 or control chaperone protein in PBS for 2 h at 55 °C. Samples were mixed with SDS loading buffer and analyzed by SDS-PAGE and Western blotting using αSyn LB509 antibody (Zymed). Quantitation of high molecular weight αSyn was performed using NIH Image J. Integrated pixel intensity of high molecular weight synuclein for each sample was normalized to monomeric synuclein intensity. For fibril formation, αSyn and chaperone proteins (as described above) were incubated with shaking for 1 wk at 37 °C. Fibril formation was assessed by Congo red (Conway et al. 2000b).

## Supporting Information

Figure S1 Additional Structural and Functional Analyses of DJ-1 In Vitro (A) DJ-1 catalase activity was quantified as compared to catalase I (5 μg/ml). DJ-1 does not display catalase activity even at concentrations as high as 5 mg/ml.
(B) Addition of DJ-1 at 5 mg/ml does not alter catalase activity of the catalase I-positive control, indicating that there are no inhibitory elements present in the DJ-1 preparation.
(C) Purity of bacterially produced DJ-1 utilized in the in vitro assays was assessed to be > 99% by SDS-PAGE and colloidal Coomassie staining.
(D) GST thermal aggregation (0.4 μM, black circles) is suppressed by WT DJ-1 (2 μM, red squares) and by positive control Hsp27 (2 μM, green stars), but not by L166P mutant DJ-1 (2 μM, blue triangles) or by RNase A (2 μM, purple diamonds).
(E) Far-ultraviolet CD spectra of WT DJ-1 (blue triangles) and the L166P mutant (red squares); mean residue ellipticity (Θ) equals °C · cm^2^ · dmol^−1^. The mutant protein displays significantly reduced secondary structure. CD spectra of DJ-1 (40 μM in 10 mM PBS [pH 7.4]) were recorded on an Aviv 62A sCD spectrometer at 4 °C in a 0.02-cm path length cuvette, and α-helix and β-sheet content were estimated as described (Sreerama and Woody 2003). Based on an initial evaluation of the spectra, the WT spectrum was analyzed using a basis set appropriate for folded proteins, whereas the mutant spectrum was analyzed using a basis set suited for unstructured proteins. Thermal stability was determined by monitoring the change in mean residue ellipticity ([Θ], equal to °C · cm^2^ · dmol^−1^) at 222 nm as a function of temperature. Thermal melts were performed in 4 °C increments with an equilibration time of 1 min and an integration time of 30 sec, using a 0.1-cm path length cuvette.
(F) Thermal denaturation curves for WT and mutant L166P DJ-1; mean residue ellipticity (Θ)_222_ is equal to °C · cm^2^ · dmol^−1^ at 222 nm.
(G) Redox regulation is unaffected by the C106A mutation. Redox regulation of C106A DJ-1 was assayed via DTT inactivation (0.5 mM) in the CS aggregation suppression assay.
(H) Protofibril preparations (as in Figure 2A and 2B, incubated for 2 h at 55 °C) do not contain Congo red–positive mature fibrils. Untreated αSyn preparations (open bars) and protofibril preparations (filled bars) were subjected to Congo red analysis as in Figure 2C.
(1.2 MB PDF).

Figure S2 Additional Studies of DJ-1 Chaperone Activity In Vivo (A) Undifferentiated ES cells were transfected with Flag-αSyn and treated with 2 mM FeCl_2_ (Fe) or media alone (0) as described in Figure 3. As expected, undifferentiated ES cultures do not express endogenous αSyn. Furthermore, the transfected Flag-αSyn does not accumulate in the Triton X-100-insoluble fraction of undifferentiated cells, in contrast to differentiated cultures.
(B) Overexpression of WT DJ-1 does not significantly alter the half-life of soluble Flag-αSyn. CAD murine neuroblastoma cells were stably transfected with Flag-tagged human α-synuclein using standard techniques. 2 × 10^5^ cells in a 24-well format were transiently transfected with eukaryotic expression constructs encoding WT human DJ-1 or empty vector. After 36 h, cells were starved for 1 h with DMEM lacking cysteine and methionine and supplemented with 8% dialyzed FBS. Cells were pulsed for 2 h with 10 μCi[^35^S]-L-Met/L-Cys (EasyTides; Perkin Elmer, Wellesley, California, United States) per well, washed twice, and chased at the indicated intervals with complete medium. Flag-αSyn was immunoprecipitated with Flag antibody-conjugated agarose beads (Sigma), subjected to SDS-PAGE, and visualized by autoradiography.
(C) Flag-αSyn from (B) was quantitated using NIH Image J.
(815 KB PDF).

Figure S3 Additional Studies of DJ-1 Mutations (A) Overexpression of WT DJ-1 or L166P DJ-1 in the context of αSyn aggregation does not alter cell number. Cells from Figure 4M were quantified via ToPro3 nuclear staining and are expressed as number of cells per field from ten independent fields in each of three wells. Data are shown as the mean ± SEM and were analyzed by ANOVA with Fisher's post-hoc test. * *p* < 0.
(B) Overexpression of WT DJ-1 or L166P mutant DJ-1 in the context of Q333P mutant NFL aggregation does not alter cell number. GFP positive transfected cells from Figure 5A–5L were quantified and are expressed as number of transfected cells per field from ten independent fields in each of three wells. Data are shown as the mean ± SEM and were analyzed by ANOVA with Fisher's post-hoc test. * *p* < 0.
(C) Overexpression of WT DJ-1, but not L166P mutant DJ-1, rescues cells from Q333P mutant NFL toxicity. HeLa cells were transfected with Q333P mutant NFL along with WT human DJ-1, L166P mutant DJ-1, or vector control. After 72 h, cells were assayed by MTT reduction assay (which detects reduction of 3-(4,5-dimethylthiazol-2-yl)-2,5-diphenyltetrazolium bromide by metabolic enzymes) (Martinat et al. 2004). Data are shown as the mean ± SEM and were analyzed by ANOVA with Fisher's post-hoc test. * *p* < 0.
(D) C53A mutant DJ-1 is unable to rescue cells from Q333P mutant NFL toxicity. Undifferentiated ES cells were transfected with Q333P mutant NFL along with WT human DJ-1, C53A mutant DJ-1, or vector control. After 72 h, cells were assayed by MTT reduction assay (Martinat et al. 2004). Data are shown as the mean ± SEM and were analyzed by ANOVA with Fisher's post-hoc test. * *p* < 0.
(E) Coexpression of DJ-1 with NFL does not alter NFL expression levels. CAD cells were transfected with Q333P mutant NFL and vector, WT DJ-1, C53A mutant DJ-1, or L166P mutant DJ-1. Cells were differentiated for 72 h and lysed to produce Triton X-100-soluble and -insoluble fractions. Lysates were exposed to Western blotting with an antibody against transfected human NFL. NFL is present only in the insoluble fraction, and expression of WT or mutant DJ-1 does not alter NFL expression levels.
(685 KB PDF).

Figure S4 DJ-1 Localization Does Not Appear Altered by FeCl_2_ Treatment CAD cells were transfected with WT DJ-1 and differentiated by serum withdrawal for 72 h. Cells were treated with medium alone (A–F) or medium with 2 mM FeCl_2_ (G–L) for 18 h prior to fixation with PFA. Cells were immunostained with rabbit anti-DJ-1 as described, followed by donkey anti-rabbit Cy5 (A, D, G, and J). Nuclei (B, E, H, and K) were visualized by incubation with the nuclear stain ToPro3 prior to imaging.
(1.6 MB PDF).

Table S1 DJ-1 Lacks Protease and Antioxidant Activities (45 KB DOC).

## Abbreviations

αSyn: α-synuclein
CD: circular dichroism
CS: citrate synthase
DN: dopamine neuron
DTT: dithiothreitol
ES: embryonic stem
Flag-αSyn: Flag epitope-tagged αSyn
GST: glutathione S-transferase
His: polyhistidine
Hsp: heat shock protein
NFL: neurofilament light subunit
PD: Parkinson's disease
ROS: reactive oxygen species
WT: wild-type
