# Supplementary figures and images for: DJ-1 Is a Redox-Dependent Molecular Chaperone That Inhibits α-Synuclein Aggregate Formation
Source: PLoS Biol. 2004 Oct 5;2(11):e362. doi: 10.1371/journal.pbio.0020362 (PMC521177; doi:10.1371/journal.pbio.0020362)

Figure S1

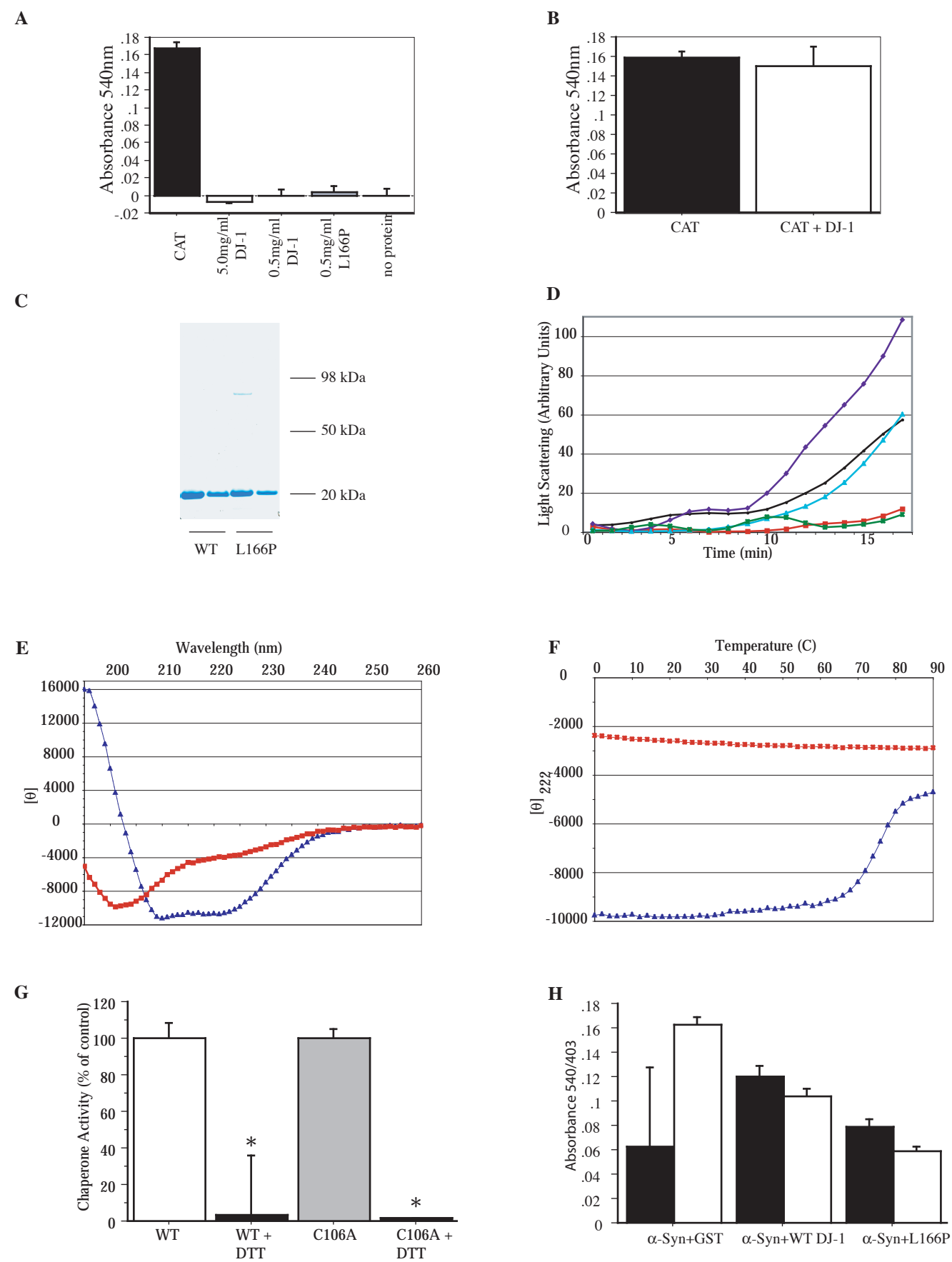

Supplement: Figure S1 — (A) DJ-1 catalase activity was quantified as compared to catalase I (5 μg/ml). DJ-1 does not display catalase activity even at concentrations as high as 5 mg/ml. (B) Addition of DJ-1 at 5 mg/ml does not alter catalase activity of the catalase I-positive control, indicating that there are no inhibitory elements present in the DJ-1 preparation. (C) Purity of bacterially produced DJ-1 utilized in the in vitro assays was assessed to be > 99% by SDS-PAGE and colloidal Coomassie staining. (D) GST thermal aggregation (0.4 μM, black circles) is suppressed by WT DJ-1 (2 μM, red squares) and by positive control Hsp27 (2 μM, green stars), but not by L166P mutant DJ-1 (2 μM, blue triangles) or by RNase A (2 μM, purple diamonds). (E) Far-ultraviolet CD spectra of WT DJ-1 (blue triangles) and the L166P mutant (red squares); mean residue ellipticity (Θ) equals °C · cm2 · dmol−1. The mutant protein displays significantly reduced secondary structure. CD spectra of DJ-1 (40 μM in 10 mM PBS [pH 7.4]) were recorded on an Aviv 62A sCD spectrometer at 4 °C in a 0.02-cm path length cuvette, and α-helix and β-sheet content were estimated as described (Sreerama and Woody 2003). Based on an initial evaluation of the spectra, the WT spectrum was analyzed using a basis set appropriate for folded proteins, whereas the mutant spectrum was analyzed using a basis set suited for unstructured proteins. Thermal stability was determined by monitoring the change in mean residue ellipticity ([Θ], equal to °C · cm2 · dmol−1) at 222 nm as a function of temperature. Thermal melts were performed in 4 °C increments with an equilibration time of 1 min and an integration time of 30 sec, using a 0.1-cm path length cuvette. (F) Thermal denaturation curves for WT and mutant L166P DJ-1; mean residue ellipticity (Θ)222 is equal to °C · cm2 · dmol−1 at 222 nm. (G) Redox regulation is unaffected by the C106A mutation. Redox regulation of C106A DJ-1 was assayed via DTT inactivation (0.5 mM) in the CS aggregation suppr [file pbio.0020362.sg001.pdf]

Figure S2

A

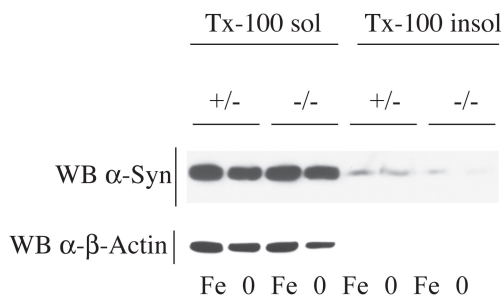

B

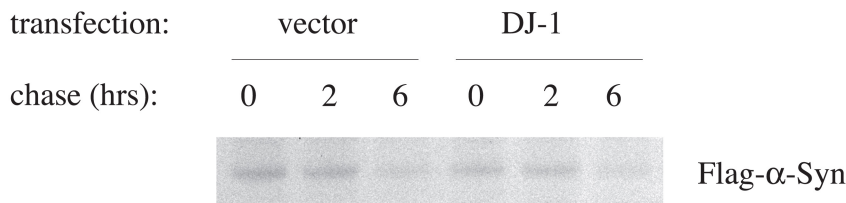

C

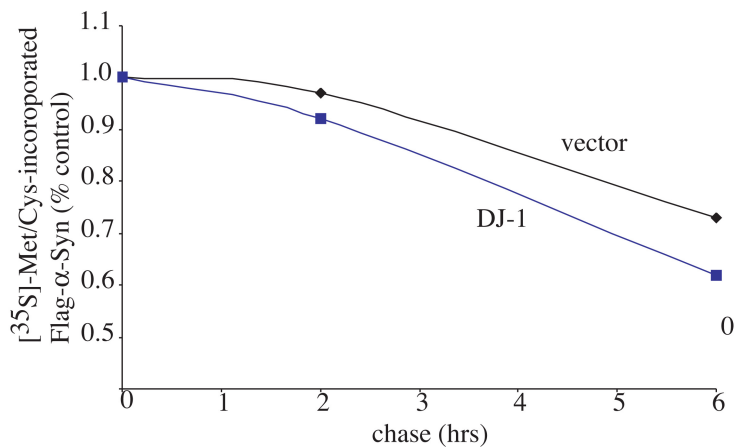

Supplement: Figure S2 — (A) Undifferentiated ES cells were transfected with Flag-αSyn and treated with 2 mM FeCl2 (Fe) or media alone (0) as described in Figure 3. As expected, undifferentiated ES cultures do not express endogenous αSyn. Furthermore, the transfected Flag-αSyn does not accumulate in the Triton X-100-insoluble fraction of undifferentiated cells, in contrast to differentiated cultures. (B) Overexpression of WT DJ-1 does not significantly alter the half-life of soluble Flag-αSyn. CAD murine neuroblastoma cells were stably transfected with Flag-tagged human α-synuclein using standard techniques. 2 × 10^5^ cells in a 24-well format were transiently transfected with eukaryotic expression constructs encoding WT human DJ-1 or empty vector. After 36 h, cells were starved for 1 h with DMEM lacking cysteine and methionine and supplemented with 8% dialyzed FBS. Cells were pulsed for 2 h with 10 μCi[35S]-L-Met/L-Cys (EasyTides; Perkin Elmer, Wellesley, California, United States) per well, washed twice, and chased at the indicated intervals with complete medium. Flag-αSyn was immunoprecipitated with Flag antibody-conjugated agarose beads (Sigma), subjected to SDS-PAGE, and visualized by autoradiography. (C) Flag-αSyn from (B) was quantitated using NIH Image J. (815 KB PDF). [file pbio.0020362.sg002.pdf]

Figure S3

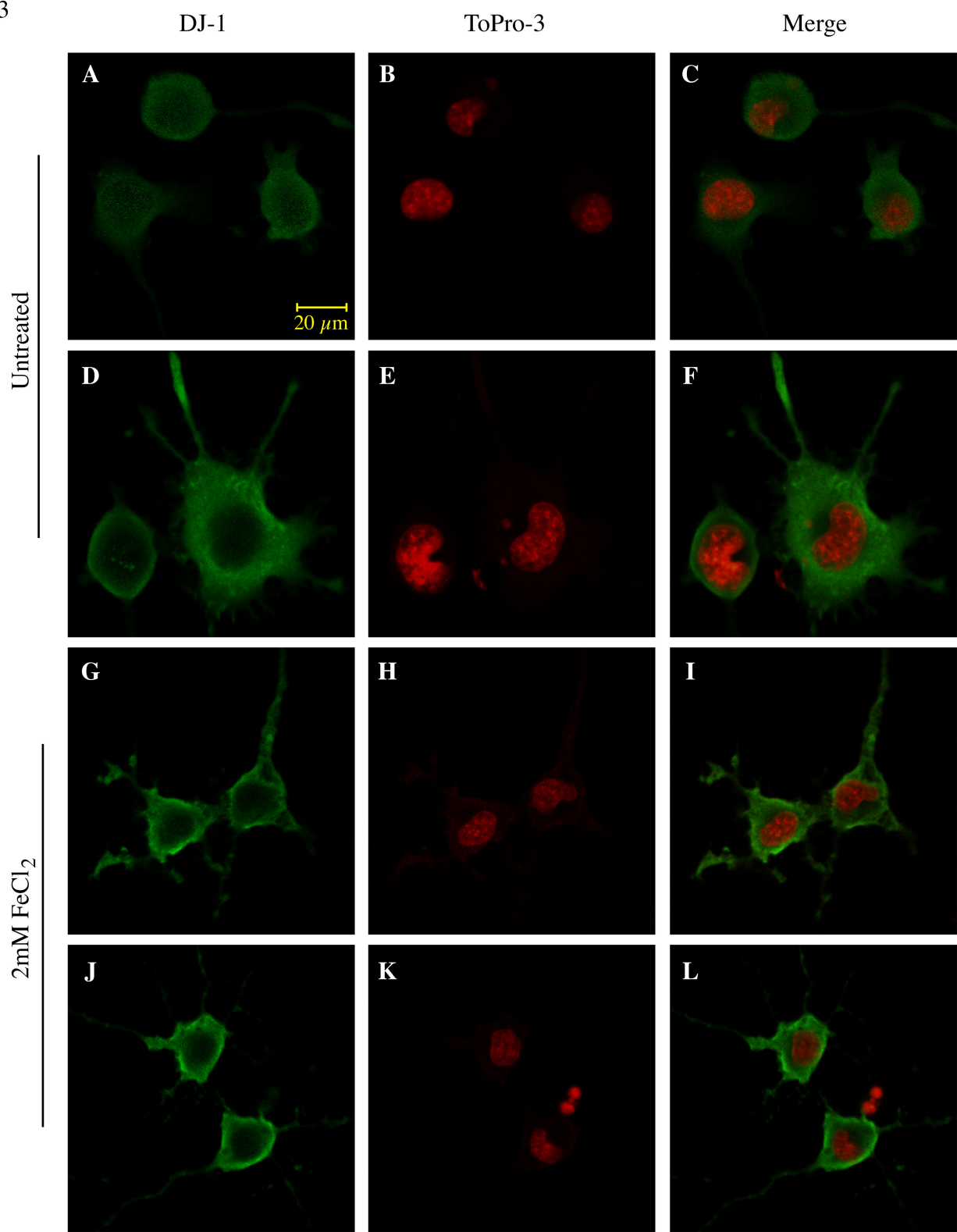

Supplement: Figure S3 — (A) Overexpression of WT DJ-1 or L166P DJ-1 in the context of αSyn aggregation does not alter cell number. Cells from Figure 4M were quantified via ToPro3 nuclear staining and are expressed as number of cells per field from ten independent fields in each of three wells. Data are shown as the mean ± SEM and were analyzed by ANOVA with Fisher's post-hoc test. * p < 0. (B) Overexpression of WT DJ-1 or L166P mutant DJ-1 in the context of Q333P mutant NFL aggregation does not alter cell number. GFP positive transfected cells from Figure 5A–5L were quantified and are expressed as number of transfected cells per field from ten independent fields in each of three wells. Data are shown as the mean ± SEM and were analyzed by ANOVA with Fisher's post-hoc test. * p < 0. (C) Overexpression of WT DJ-1, but not L166P mutant DJ-1, rescues cells from Q333P mutant NFL toxicity. HeLa cells were transfected with Q333P mutant NFL along with WT human DJ-1, L166P mutant DJ-1, or vector control. After 72 h, cells were assayed by MTT reduction assay (which detects reduction of 3-(4,5-dimethylthiazol-2-yl)-2,5-diphenyltetrazolium bromide by metabolic enzymes) (Martinat et al. 2004). Data are shown as the mean ± SEM and were analyzed by ANOVA with Fisher's post-hoc test. * p < 0. (D) C53A mutant DJ-1 is unable to rescue cells from Q333P mutant NFL toxicity. Undifferentiated ES cells were transfected with Q333P mutant NFL along with WT human DJ-1, C53A mutant DJ-1, or vector control. After 72 h, cells were assayed by MTT reduction assay (Martinat et al. 2004). Data are shown as the mean ± SEM and were analyzed by ANOVA with Fisher's post-hoc test. * p < 0. (E) Coexpression of DJ-1 with NFL does not alter NFL expression levels. CAD cells were transfected with Q333P mutant NFL and vector, WT DJ-1, C53A mutant DJ-1, or L166P mutant DJ-1. Cells were differentiated for 72 h and lysed to produce Triton X-100-soluble and -insoluble fractions. Lysates were exposed to Western blotting with an antibody [file pbio.0020362.sg003.pdf]

Figure S4

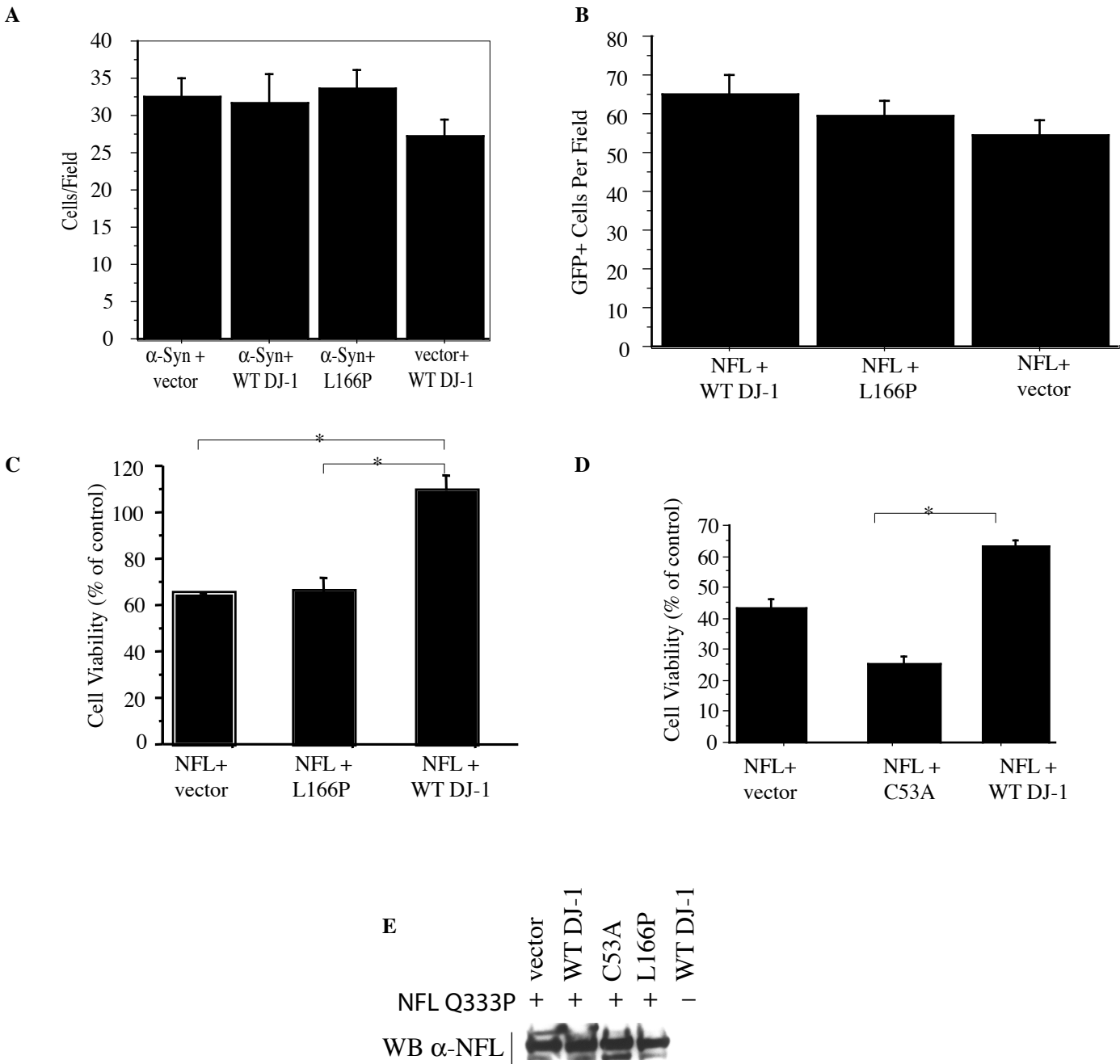

Supplement: Figure S4 — CAD cells were transfected with WT DJ-1 and differentiated by serum withdrawal for 72 h. Cells were treated with medium alone (A–F) or medium with 2 mM FeCl2 (G–L) for 18 h prior to fixation with PFA. Cells were immunostained with rabbit anti-DJ-1 as described, followed by donkey anti-rabbit Cy5 (A, D, G, and J). Nuclei (B, E, H, and K) were visualized by incubation with the nuclear stain ToPro3 prior to imaging. (1.6 MB PDF). [file pbio.0020362.sg004.pdf]
