# Supplementary material for: DJ-1 Is a Redox-Dependent Molecular Chaperone That Inhibits α-Synuclein Aggregate Formation
Source: PLoS Biol. 2004 Oct 5;2(11):e362. doi: 10.1371/journal.pbio.0020362 (PMC521177; doi:10.1371/journal.pbio.0020362)
Supplement: Table S1 — (45 KB DOC). [file pbio.0020362.st001.doc]

**SUPPLEMENTARY TABLE 1.** Purified DJ-1 does not exhibit protease or antioxidant activities in several assays.

| **Assay** | **Activity** | **Substrate** | **Negative Control**  **(no protein)** **Arbitrary Units (AU)** | **Positive Control**  **(cell lysate)**  **(AU)** | **DJ-1**  **(AU)** |
| --- | --- | --- | --- | --- | --- |
| Fluorogenic Protease Assay | Chymotrypsin/ tripeptidyl peptidase | AAFAMC  (Balow et al., 1986) | 30845  SEM=530 | 284319  SEM=1156 | 31123  SEM=173 |
|  | Cysteine-Protease | YVAD-AMC (Thornberry et al., 1992) | 46491  SEM=813 | 57313  SEM=2020 | 46406  SEM=2096 |
|  | Chymotrypsin | LLVY-AMC (Garcia-Calvo et al., 1999) | 3414  SEM=18 | 63047  SEM=741 | 3693  SEM=27 |
| Fluorogenic Protease Assay  5mM ATP, 10mM MgCl_2_ | Chymotrypsin/ tripeptidyl peptidase | AAF-AMC | 26345  SEM=2413 | 127393 SEM=3867 | 28618  SEM=6417 |
|  | Cysteine-Protease | YVAD-AMC | 41488  SEM=397 | 51478  SEM=495 | 43430 SEM=408 |
|  | Chymotrypsin | LLVY-AMC | 3375  SEM=17 | 40670  SEM=375 | 3366 SEM=23 |
| Gel Overlay Assay | Cys/Ser protease (Halio et al., 1996) | Gelatin | Inactive | Active | Inactive |

| Glutamine Synthase Protection Assay | Antioxidant  (Spatafora et al., 2002) | Glutamine Synthetase | .0785  SEM=.0065 | .1795 SEM=.0075 | .0660 SEM=.0061 |
| --- | --- | --- | --- | --- | --- |

**METHODS**

Protease activity of DJ-1 was investigated using three fluorogenic substrates: Suc-AAF-amido-4-methylcoumarin (AMC), an amino-endopeptidase substrate; Suc-LLVY-AMC, a chymotrypsin-like substrate; and Suc-YVAD-AMC, a caspase substrate. Release of AMC from the fluorogenic peptide substrates results in an increase in fluorescence of the solution over time if the specified type of protease is present. The assays were performed in two buffers in parallel. Buffer 1 contained only 50mM Tris, pH7.6, 100mM NaCl. Buffer 2 contained 50mM Tris, pH7.6, 100mM NaCl, 5mM ATP, 10mM MgCl2, and 1mM DTT. Cleared bacterial cell lysate was used as a positive control for protease activity, and buffer alone was used as a negative control. Both WT and L166P DJ-1 were tested in these assays at a concentration of 300μg/ml. Protease activity was measured at both room temperature and 37^o^C, and we failed to detect DJ-1 activity in either case. Similar results were obtained using the succinylated Casein assay (Pierce), a general protease assay supplemented with CaCl2, ATP, or both CaCl2 and ATP.

For the gel overlay assay, bacterially produced DJ-1 and Flag-DJ-1 purified from Flag-DJ-1 expressing stable CAD cells were run on Tris-Glycing SDS-PAGE gels containing 1% Collagen. Gels were then renatured and incubated at 37C to allow proteolytic activity to occur. Gels were then stained with Coomassie to visualize the Collagen and were inspected for cleared areas that would indicate protease activity and degredation of the collagen substrate. Cleared bacterial lysate was used as a positive control. No proteolytic activity was seen for any preparation of DJ-1.

The glutamine synthase protection assay was performed essentially as described (Spatafora et al., 2002). Similarly, no catalase activity was detected using a commercial catalase assay (Cayman).

**References**

Balow, R. M., Tomkinson, B., Ragnarsson, U., and Zetterqvist, O. (1986). Purification, substrate specificity, and classification of tripeptidyl peptidase II. J Biol Chem *261*, 2409-2417.

Garcia-Calvo, M., Peterson, E. P., Rasper, D. M., Vaillancourt, J. P., Zamboni, R., Nicholson, D. W., and Thornberry, N. A. (1999). Purification and catalytic properties of human caspase family members. Cell Death Differ *6*, 362-369.

Halio, S. B., Blumentals, II, Short, S. A., Merrill, B. M., and Kelly, R. M. (1996). Sequence, expression in Escherichia coli, and analysis of the gene encoding a novel intracellular protease (PfpI) from the hyperthermophilic archaeon Pyrococcus furiosus. J Bacteriol *178*, 2605-2612.

Spatafora, G., Van Hoeven, N., Wagner, K., and Fives-Taylor, P. (2002). Evidence that ORF3 at the Streptococcus parasanguis fimA locus encodes a thiol-specific antioxidant. Microbiology *148*, 755-762.

Thornberry, N. A., Bull, H. G., Calaycay, J. R., Chapman, K. T., Howard, A. D., Kostura, M. J., Miller, D. K., Molineaux, S. M., Weidner, J. R., Aunins, J., and et al. (1992). A novel heterodimeric cysteine protease is required for interleukin-1 beta processing in monocytes. Nature *356*, 768-774.
